# Vocal imitation of percussion sounds: On the perceptual similarity between imitations and imitated sounds

**DOI:** 10.1371/journal.pone.0219955

**Published:** 2019-07-25

**Authors:** Adib Mehrabi, Simon Dixon, Mark Sandler

**Affiliations:** 1 Department of Linguistics, Queen Mary University of London, London, England; 2 School of Electronic Engineering and Computer Science, Queen Mary University of London, London, England; University of Birmingham, UNITED KINGDOM

## Abstract

Recent studies have demonstrated the effectiveness of the voice for communicating sonic ideas, and the accuracy with which it can be used to imitate acoustic instruments, synthesised sounds and environmental sounds. However, there has been little research on vocal imitation of percussion sounds, particularly concerning the perceptual similarity between imitations and the sounds being imitated. In the present study we address this by investigating how accurately musicians can vocally imitate percussion sounds, in terms of whether listeners consider the imitations ‘more similar’ to the imitated sounds than to other same–category sounds. In a vocal production task, 14 musicians imitated 30 drum sounds from five categories (cymbals, hats, kicks, snares, toms). Listeners were then asked to rate the similarity between the imitations and same–category drum sounds via web based listening test. We found that imitated sounds received the highest similarity ratings for 16 of the 30 sounds. The similarity between a given drum sound and its imitation was generally rated higher than for imitations of another same–category sound, however for some drum categories (snares and toms) certain sounds were consistently considered most similar to the imitations, irrespective of the sound being imitated. Finally, we apply an existing auditory image based measure for perceptual similarity between same–category drum sounds, to model the similarity ratings using linear mixed effect regression. The results indicate that this measure is a good predictor of perceptual similarity between imitations and imitated sounds, when compared to acoustic features containing only temporal or spectral features.

## Introduction

Ethnographic studies with music producers have shown that searching for short sounds such as drum samples is a core part of the electronic music making process, yet current search methods such as manually navigating audio files leave users frustrated and unable to efficiently find the sounds they seek [1]. This highlights a need for audio search and retrieval systems that provide an expressive means for capturing and translating the sonic qualities that the user is looking for in a target sound. The voice is an attractive medium for this as it can be used to express timbral, tonal and dynamic temporal variations [2], providing one of the most versatile tools at our disposal for expressing sonic ideas. This versatility is commonly exploited by musicians to communicate, teach and perform non–verbal musical sounds [3]. Previous studies on non–verbal vocalisations have demonstrated the effectiveness of vocal imitations for describing and communicating sounds [4, 5], and highlighted the ability of musicians to accurately imitate musical sounds with respect to basic acoustic features [6, 7]. In addition, previous research on vocal imitations has shown that human listeners are able to identify the sound being imitated with above chance accuracy for acoustic instruments, synthesised sounds, and everyday (environmental) sounds [8]. However, to date there has been little research focussing on the vocal imitation accuracy and imitability of percussion sounds.

Query by vocalisation (QBV) is the process of searching for sounds based on vocalised examples of the desired sound. Typically, in QBV systems acoustic features are extracted from a vocal example of a target sound and compared to the features of sounds in an audio library, to return a ranked list of similar sounds. Such systems are typically evaluated based on the accuracy with which vocalisations are matched to class labels [9], or vocal imitations are matched to the imitated sounds [10–12]. Whilst this may be a valid assumption for certain tasks, we hypothesise that the imitated sound (or indeed class label) may not always be the most perceptually similar sound (or class) to the vocalisation. In such a case, the imitated sound (or class) should not be ranked highest in the search results. In order to retrieve sounds based on perceptual similarity, we require models of vocalisations and audio samples that are based on similarity ratings provided by human listeners. The core motivations of this work are to establish whether imitated sounds are indeed always considered most similar to the respective imitations by listeners, and to investigate methods for modelling and predicting these similarity ratings.

We address this by conducting a two part study. In the first part, a group of 14 musicians were tasked with imitating 30 drum sounds from 5 drum categories. In the second part, listeners were asked to rate the similarity between the imitations and same–category drum sounds. In doing so, we establish whether musicians can vocally imitate percussion sounds such that third party listeners consider the sounds being imitated as more similar to the imitations than other same–category sounds. The contributions of this work are threefold: i) results of the vocal imitation and listening study are presented, demonstrating the degree to which musicians can vocally imitate percussion sounds; ii) we evaluate an existing model for perceptual similarity between same–category drum sounds in terms of its suitability for modelling similarity between vocal imitations and same–category percussion sounds. This is compared to a set of acoustic descriptors commonly used in music information retrieval and speech analysis; iii) we make available to the research community a dataset of 420 vocal imitations, 30 drum sounds, and 11340 perceptual similarity ratings between the imitations and drum sounds.

## Related work

Measuring the perceptual similarity between sounds has received a great deal of attention from the music information retrieval (MIR) and music perception communities. Much of this work has been concerned with modelling the perceptual similarity of instrument timbres using acoustic descriptors (see [13] and [14] for an overview of the methods used for such tasks). Typical methods for measuring sound similarity from listener responses include asking listeners to rate similarity between pairs of sounds [15, 16], ranking (or rating) the quality of a set of sounds in relation to a reference sound [17], and manually sorting sounds into groups or clusters [18, 19]. These similarity measures can be used to derive a Euclidean space in which the distance between sounds is proportional to their perceptual similarity, using techniques such as multidimensional scaling. Whilst there have been many such studies focussing on sound–wise similarity of acoustic instruments, there is scarce research on similarity measures between vocal imitations and acoustic instruments, in particular for drum sounds. That said, there have been a number of relevant studies concerning both vocalised percussion sounds and vocal imitations of more general sounds, which we discuss in this section.

One of the most prominent cases of vocalised percussion sounds is the art form of beatboxing: a vocal performance technique where the performer imitates percussion sounds and rhythmic patterns (see [20] for an overview of techniques that characterise the practice). Although it is outside the scope of this work, the similarities between beatboxing and the present study warrant a brief discussion of the related literature. Proctor et al. [21] examined the mechanisms that a professional hip hop vocalist and beatboxer used to imitate percussion sounds, using real-time magnetic resonance imaging. The beatboxer demonstrated use of articulation and airstream mechanisms found in speech, but also used articulation patterns that did not exist in their native language, indicating that when imitating percussion sounds people may use vocal techniques beyond those found in speech. Lederer [22] investigated imitation accuracy of six popular electronic drum sounds from a sole beatboxer. She found that beatboxed sounds contained more frequency components than their electronic drum counterparts and that the temporal envelopes were not accurately imitated, particularly for transient sounds such as clicks, where the vocalist had little or no control over the decay portion of the sound. In general, electronic sounds were identifiable from the vocalisations and it was noted that the more degrees of freedom one has over a sound (in terms of vocal production), the less accurate the imitation is (i.e. hi–hats were imitated less accurately than clave clicks). There have also been a number of studies on the automatic classification of beatboxed sounds for determining drum categories [23, 24] and mapping to drum sounds [25], although these do not incorporate measures of perceptual similarity between the vocalisations and actual drum sounds. We note that in the present study we did not specifically recruit beatboxers, and in general the imitators did not have previous experience of vocal imitation practice (although one of the 14 imitators was an experienced beatboxer).

Verbally describing sounds can be difficult, particularly when the source of the sound is unknown or when trying to describe the differences between two sounds from similar sources. Previous studies have shown that vocal imitations can be as good or better than verbal descriptions for identifying the cause of a sound, based on imitations of everyday, complex, artificial and mechanical sounds [4, 5]. Although similarity was not directly measured, Lemaitre and Rocchesso [5] found that participants were generally able to identify an imitated sound from a set of nine possible sounds, based on a single imitation. The authors noted that recognition accuracy was reduced when the frequency range of the imitations was smaller than that of the imitated sounds, and when the imitated sounds contained temporal variations that were not easy to vocalise. This demonstrates the effectiveness and some limitations of accurately imitating non–verbal sounds.

Vocal imitation has received considerable attention in the linguistics literature, featuring as a mechanism for language learning [26, 27], and is closely related to the concept of sound symbolism—that linguistic sounds such as phonemes might be acoustically related to word meaning, at least for certain groups of words [28]. Beyond words, non-verbal vocal imitation can serve as a powerful means of communication and an integral part of language, seen for example in the language of the Mbendjele Pygmies in the Congo basin [29]. The Mbendjele employ vocal imitation of environmental sounds such as animal calls and sounds of the forest for a number of purposes, including hunting and play, and Lewis notes that such diverse methods of communication may have played a key role in the survival of early humans. In addition, there has been recent work on the iconicity and word-likeness of vocal imitations, where imitations of environmental sounds were repeatedly imitated for 8 ‘generations’, along a chain of ‘speakers’ and listeners [30]. This process resulted in imitations that were more stable and word-like (in terms of the imitations in latter generations sounding more similar to one-another than those from earlier generations) whilst retaining salient acoustic characteristics that carry information about the sounds that were originally imitated. Although the present work is concerned with vocal imitation of music-related sounds, the general question of what makes particular sounds more or less imitable than others, and studying the acoustic characteristics that allow for the effective communication of sonic ideas, has much broader applications in furthering our understanding the role of vocal imitation in language.

Sitting between words and such non-verbal vocal imitations are onomatopoeia. Onomatopoeia are a special class of words that fit clearly within the realm of imitation, being vocal representations of sounds in the world around us. Their phonemic make up does not appear to follow the typical patterns of non-onomatopoeic words. For example, it has been shown for English that the frequency of sounds in onomatopoeia varies considerably to that found in the lexicon—with onomatopoeia containing more consonants and stops, yet fewer fricatives [31], and there is evidence suggesting that the neural processing of onomatopoeia employs faculties required for both verbal and non-verbal processing [32]. As such, onomatopoeia present an interesting area for research on the relation between vocal imitation and language. However, as Lemaitre and Rocchesso note, there is a reasonable body of literature that has investigated the use of onomatopoeia, whilst the study of non-conventional vocal imitation has received much less attention [5]. In addition, onomatopoeia provide a prototypical generalisation of a concept (be it an object, animal, or physical action). This may be considered an iconic representation that has obvious uses in language (for example, using the word ‘bang’ to represent a drum sound), but may contain very little information regarding the specificities of the particular sound being imitated (such as the sound of a specific drum being stuck by a musician in a particular manner). For this reason, as with previous studies on non-conventional vocal imitation, the use of onomatopoeia and symbolic representations are not investigated in the present work, where we are interested in the ability of musicians to accurately imitate individual sounds.

Regarding listener–based classification of vocal imitations, Dessein and Lemaitre [33] asked listeners to freely sort vocal imitations into clusters, and found that they tended to discriminate between perceptual categories based on the sound source of the imitated sounds (such as gas, liquid, solid). In another sorting task, Rocchesso et al. asked listeners to sort vocal imitations into clusters based on similarity to a referent sound from each cluster [34]. The imitations were from a sole voice artist, and the authors found that approximately 50% of the sounds were sorted into the same clusters as those determined by a computational clustering method (using basic acoustic features, and k–means clustering in a PCA-reduced 2–dimensional feature space). Although these studies show the ability of listeners to group vocal imitations into meaningful clusters based on timbral similarity or the source of the sounds, they do not consider the similarity between vocal imitations and same–class sounds.

In addressing this, Cartwright and Pardo asked listeners to identify the sound being imitated from a set of 10 sounds in a forced–choice experiment [8]. They found recognition accuracy was highest for environmental sounds (80%), and lower, but still above chance for acoustic instruments (45%) and synthesised sounds (42–54%). The authors also released a large dataset of vocal imitations and listener responses from this study, providing an excellent resource for research on QBV methods. One can infer the ability of humans to imitate certain types of sounds based on the results from the forced–choice task, however this experimental design does not provide information regarding the perceptual similarity between imitations and each of the imitated sounds. Collecting such measures would be resource intensive given the large number of imitated sounds and imitations provided in this dataset. As such, in the present study we focus on one type of sound (percussion), using a much smaller set of sounds (30), and limit the scope to measuring similarity between imitations and same–category drum sounds (i.e. kick, snare etc.).

The accuracy of vocal imitations has also been investigated in terms of acoustic features [6, 7, 35]. Assuming that the acoustic features are perceptually relevant, one might infer that a more accurate imitation (in terms of the acoustic features) is also more similar to the imitated sound. In previous work [7] we showed that musicians were able to accurately imitate the envelopes of pitch, loudness and spectral centroid features, for simple synthesised tones based on single features or combinations of 2 features. In addition, it has been demonstrated that participants are able to accurately imitate pitch, tempo and sharpness features, yet are unable to differentiate their imitations in terms of subtle differences in attack times [6]. Furthermore, both heuristic (hand–crafted) and learned acoustic features have been used to map between imitations and imitated sounds for the purposes of QBV and classifying vocal imitations [9, 10, 12, 36, 37]. Features learned using deep learning techniques have been shown to outperform Mel frequency cepstral coefficients (MFCCs) [38] for retrieving imitated sounds [10, 12], and features that represent the signal shape and time evolution of sounds perform well at classifying imitations in terms of their morphological profile [36]. However, it is not known how well the features used in these studies correlate with listeners’ perception of similarity between imitations and the imitated sounds.

Finally, Lemaitre et al. [35] investigated whether acoustic features could be used to predict listener–based classification accuracy of vocal imitations. Listeners were asked to classify vocal imitations in a two–way forced choice experiment (i.e. given a vocalisation, state whether it is an imitation of a ‘fridge’ or ‘blender’). Two sets of acoustic features were compared: one based on Euclidean distance between sounds in a feature space derived using morphological descriptors from [36], and one based on the alignment cost between the spectrograms of two sounds (using dynamic time warping). The spectrogram alignment cost outperformed the morphological descriptors in terms of predicting listener classification accuracy. Most notably, the authors highlight that the correlation of distance (in terms of alignment cost) over classification accuracy varied considerably across the different families of imitated sounds (such as impulsive, stationary, and complex sounds), suggesting that the acoustic descriptors required to predict the similarity between a vocal imitation and sound class may be specific to the type of sound being imitated.

The work discussed in this section demonstrates the ability of humans to imitate a broad range of sounds using their voice. This research has focussed on i) clustering imitations into meaningful categories, ii) communication of sounds via vocal imitation, iii) identifying the imitated sound from an imitation, and iv) measuring imitation accuracy in terms of acoustic features. These findings provide a strong case for using the voice as a tool for audio search and retrieval. Nonetheless, we note two key areas for further research that are investigated in the present study: 1) The types of sounds used in these studies are typically environmental, everyday and synthesised sounds. If we wish to apply QBV to music production tasks, further work is required that focusses on the types of sounds typically used in this domain, such as drum sounds. The authors are not aware of any work to date that investigates how effectively drum sounds can be communicated using vocal imitation, in terms of the perceptual similarity between vocalisations and drum sounds. This is the focus of the initial experiments presented herein. 2) methods for QBV are typically evaluated using vocal imitation datasets [10, 12, 36] where the aim is to retrieve the imitated sound. It is convenient to evaluate such systems based on the assumption that the target sound is indeed the sound that was imitated, however there may exist other sounds in the dataset that are perceptually more similar to the vocalisation than the sound being imitated. This is particularly relevant for applications of such systems in a music production context, where a user may have an idea of the type of sound they want to retrieve, as opposed to a specific sound that they know exists in the dataset. In this case one might be looking for a sound with certain characteristics, and want to browse a range of sounds that meet this criteria based on a vocal query. We therefore argue that in order to evaluate the performance of QBV systems, we require a model of perceptual similarity between vocalised sounds and the types of sounds in a given database, not simply between the imitations and imitated sounds. This is addressed in the latter experiments of this work, where a number of acoustic features are investigated for predicting the perceptual similarity ratings between imitations and a set of same–category sounds.

## Methodology

### Data collection (vocal imitations)

The vocal imitations used for the listening study were recorded by 14 participants, each of whom vocalised the same 30 drum sounds (420 imitations). The drum sounds were split into five drum categories (cymbals, hats, kicks, snares, toms), with six sounds in each category. In this section we describe the methods used to select the drum sounds and record the imitations.

#### Selecting the drum sounds

The drum sounds were taken from the FXpansion *BFD3 Core* and *8BitKit* sample libraries [39]. These libraries contain high quality recordings of classic acoustic drums (such as Mapleworks and Bosphorus) and popular electronic drum machines (such as the Roland TR808 and Roger Linn LM–1). This gave 447 unique drum recordings, which was too many sounds for a participant to imitate. We therefore selected a subset of six samples from each category.

To make the selection we used the *auditory image* based drum similarity method from [40], which the authors show to be highly correlated with perceptual ratings of similarity for same–category drum samples (note: we use the term *auditory image* in keeping with the authors’ description). In summary, similarity between two drum sounds is measured as the Euclidean distance between their vectorised auditory images, constructed from a spectrogram with the following parameters: 93ms window; 11.6ms hop; Bark scale (72 bins); loudness in dB and scaled using Terhardt’s model for the outer and middle ear [41]. The auditory images are time aligned, and where two sounds are not of the same length the shorter image is zero padded to the length of the longer one.

A diverse range of sounds from each drum category were selected as follows. For each category, we first selected a random seed sample (*S*_1_). We then selected the most and least similar samples to *S*_1_ (*S*_2_ and *S*_6_ respectively). Finally, we selected samples *S*_3_, *S*_4_, *S*_5_ such that they were equally spaced in distance between *S*_2_ and *S*_6_, with respect to *S*_1_. This gave six samples spanning the range from most to least similar to the seed sample. We note that this method does not guarantee the most diverse selection of samples will be selected from a category, and the selected samples are dependent on the seed. However, in practice we found that the selected samples spanned a broad range of the sounds in each category. The samples are summarised in Table 1. The duration of the stimuli varies between categories, with the cymbals and some of the hats having quite long durations (up to 25.42s), whilst the kicks, snares, and toms have shorter durations more typical of drum sounds. However, it should be noted that because the stimuli come from professional sample libraries, the full extent of the decay has been included in the recording. As such, the stimuli with longer durations contain extremely long low amplitude decays, following short, high-energy attack sections. For example, in *cymbal6*, the root mean squared (RMS) power for the first 3s of the recording is 23dB higher than the RMS power for the remaining 22.42s of the decay.

**Table 1:** Selected drum samples used as stimuli for the vocal imitations. Descriptions and articulations are taken from the sample library documentation and are not exhaustive desciptions of the recording setup, strike style or drum machine settings etc. Unless specified otherwise the acoustic drums were struck with a stick.

Stimulus	Drum	Articulation	Duration (s)
cymbal1	Paiste 2002 Power Bell Ride	bow	20.96
cymbal2	Paiste Signature Dry Heavy Ride	edge	15.40
cymbal3	Bosphorus China 20	mallet, edge	9.68
cymbal4	Bosphorus Splash 10	mallet, bell	4.83
cymbal5	Paiste Signature Full Crash	bell	5.93
cymbal6	Paiste 2002 Power Bell Ride	edge	25.42
hat1	Paiste Signature	closed	0.63
hat2	Sequential Circuits DrumTraks	closed	0.13
hat3	Zildjian New Beats-Mastersound	half open	2.22
hat4	Linn LM-1	open	1.44
hat5	Paiste 2002	open	10.27
hat6	Bosphorus	brush, half open	5.69
kick1	DW Mardi Gras Sparkle	kick in (mic)	0.68
kick2	DW Mardi Gras Sparkle	kick out (mic)	0.89
kick3	Gretsch Purple	kick out (mic)	0.92
kick4	Linn LM-1	–	0.41
kick5	Mapleworks Custom	kick in (mic)	0.94
kick6	Roland 808	long duration	0.60
snare1	Roland 909	medium duration	0.38
snare2	Oberheim DX	medium duration	0.23
snare3	Roland 909	short duration	0.22
snare4	Ludwig Hammered Supraphonic	half edge	2.52
snare5	Tama Bell Brass	full hit	1.01
snare6	Tama Bell Brass	half edge	0.99
tom1	Gretsch Purple High	rim	1.45
tom2	Gretsch Purple Mid	rim	1.39
tom3	Mapleworks Custom Floor	mallet, full hit	2.49
tom4	Mapleworks Custom Mid	rim	3.24
tom5	Sequential Circuits DrumTraks	–	1.65
tom6	Mapleworks Custom Mid	brush, rim	3.31

#### Participants

The 14 participants (herein referred to as the imitators) were musicians and music producers, all of whom reported no hearing or speech impairments. The age range was 26–43 and the median age was 30.5. One female took part in the study, and the remaining imitators were male. We note that the severe sex imbalance means we cannot generalise our results across the sexes, however as we were not concerned with testing any effects of sex we decided not to exclude imitations from the female imitator in the listening study.

The imitators took part in the study during June 2016, and were recruited through professional and personal networks. Informed, written consent was provided by all participants, and the experiment was approved by the research ethics committee of Queen Mary University of London (Ref. QMREC1491). All imitators were experienced in making music or producing music containing drum samples on a computer, and reported having more than 5 years’ experience playing an instrument. Three imitators reported experience in singing but stated the voice was not their sole or main instrument. One imitator was an experienced beatboxer (> 5 years’ experience). All imitators reported having more than 2 years’ experience making music using synthesisers and/or samplers, and eleven had more than 5 years’ experience doing this.

#### Procedure

The imitation recording procedure followed the same method as that developed in [7]. The study took place in an acoustically treated and sound deadened room (Media and Arts Technology Studios, Queen Mary University of London). The recording chain was an AKG C414 (AKG Acoustics, Austria) microphone with cardioid polar pattern, low cut disabled, and no pad engaged with an Apogee Duet 2 (Apogee Electronics, CA) audio interface (microphone preamp and analogue to digital converter). The microphone was held in a shock mount and placed on a microphone stand in a fixed position. The imitators were positioned approximately 30cm from the capsule of the microphone, on axis, and instructed to maintain this distance during the recordings. The monitoring chain was an Apogee Duet 2 interface and PMC AML (PMC, England) monitors. All audio was recorded at a sampling rate of 44.1kHz and bit depth of 24. The imitators were seated at a computer and presented with a basic interface for auditioning the drum sounds and recording their imitations. They were advised that the aim of the study was to listen to each drum sound and imitate it as accurately as possible using their voice, avoiding the use of onomatopoeia or words. The lead researcher then gave an overview of the interface and left the room for the duration of the study.

The order of the drum sounds was randomised. Each drum sound could be auditioned and rehearsed as many times as the imitator wanted. The imitators were not able to listen back to their recordings, however if they were not happy with their performance they were able to re–record it as many times as they wished before proceeding to the next drum sound. The imitators were advised that the final recording of each sound would be used for the analysis.

### Listening study

A listening study was then conducted where participants were asked to rate the perceptual similarity between the vocal imitations and drum sounds. The stimuli and format of the listening study are described in this section.

#### Participants

The participants (herein referred to as the listeners) were recruited from professional and research networks (predominantly electronic mailing lists) between July and September 2016. Informed consent was provided by all participants via a web form as part of the listening test website, and the experiment was approved by the research ethics committee of Queen Mary University of London (Ref. QMREC1717a). Of the 63 participants, 46 were male and 16 were female (one chose to not disclose their sex). The age range was 18–66, with a median age of 30. All listeners reported no hearing impairments.

#### Stimuli and procedure

The 420 vocal imitations and 30 drum sounds were used as stimuli. For a given imitation, listeners rated the similarity between the imitation and the six same–category drum sounds, using a format based on the MUSHRA protocol for subjective assessment of audio quality [17]. Typically the MUSHRA format requires that a single known reference sound is compared to up to 14 test sounds, which include a hidden reference and (optional) hidden anchor. When used to judge audio quality (for which the standard was originally intended), the listener rates the audio quality of each test sound in relation to the reference. The hidden reference and anchor serve to ensure the listener is able to identify an obvious ‘best’ and ‘worst’ case example and uses the full range of the rating scale.

In the present study the known reference was an imitation, and the six test sounds were the sounds from the same drum category as the imitation. Instead of audio quality, listeners rated the test sounds in terms of similarity to the imitation. The imitated sound can be considered a hidden reference, however we did not necessarily expect this to be rated as the most similar test sound, particularly if an imitation sounded more similar to one of the other drum sounds. This test format allowed us to determine whether an imitation was similar enough to the imitated sound such that an independent listener could identify the imitated sound. As has been previously noted by Sporer et al., it also provides an inherent ranking of similarity for each of the drum sounds with respect to the imitation [42]. Finally, whilst the MUSHRA standard specifies the use of expert listeners, it has recently been shown that for assessment of source separation audio quality, lay listeners can provide comparable results to expert listeners [43].

**Fig 1:**
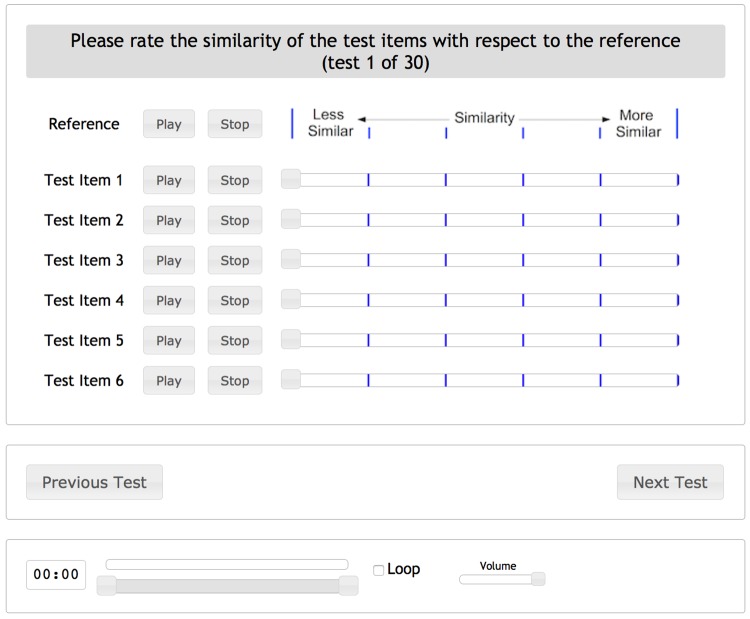
Graphical interface of a single test page used for the online listening test. Listeners were asked to rate the similarity between the imitation (reference) and 6 test items (same–category drum sounds), on a continuous scale from ‘less similar’ to ‘more similar’.

The experiment was conducted remotely via a webpage built using the BeaqleJS framework [44]. Each imitation was presented on a separate test page (trial), made up of a reference (imitation) and six ‘test’ items (drum sounds), presented in a random order. Due to the large number of imitations, each listener only rated a random subset of 28 randomly ordered imitations plus two random duplicate imitations, giving 30 trials each. The duplicate test pages were included to assess the intra–rater reliability of each participant. The listeners were instructed to rate the similarity of the test items with respect to the reference, using continuous unnumbered sliders from ‘less similar’ to ‘more similar’ (see Fig 1). These ratings were recorded on a scale from 0—1. It was possible to navigate forward and backward through the test pages, adjust volume and loop the samples. The study took approximately 30 minutes to complete.

## Results and discussion

Each listener provided 180 similarity ratings (six responses per test page, 30 test pages). There were 63 listeners in total, giving 1890 test pages and 11340 responses. In every test page there was a notable difference between the most and least similar drum sounds. If the listener gave the same rating to all six sounds, this would indicate that the listener had not followed the instructions properly; hence such test pages were removed. There were 80 invalid test pages (480 responses). The majority of these were from three listeners (25, 22 and 15 each). The web server was configured to maintain a balanced distribution of completed valid test pages per imitation: 408 imitations were rated four times and 12 were rated five times, excluding the duplicate test pages.

### Intra–rater reliability

Listener reliability was assessed using the Spearman rank correlation between the two duplicate test pages for each listener. We used the ranks because we expected some variability due to the continuous response scale, and were mainly interested in whether participants could replicate the ordering of their responses, not the exact rating values. Reliable listeners were defined as those who were able to replicate their responses for at least one of the duplicates with *ρ* >= 0.5. We note that this value is somewhat arbitrary, but it indicates a large positive correlation [45] hence was deemed suitable for the purposes of identifying unreliable listeners. There were 51 reliable listeners, for whom *ρ* = 0.63/0.04 (mean/standard error), giving 9126 responses from 1521 test pages (excluding duplicates).

### Concordance of ratings (inter–rater agreement)

Concordant imitations are those for which there was agreement amongst the listeners regarding the similarity ratings. We computed Kendall’s coefficient of concordance [46] on the ranked ratings for each imitation, excluding those from unreliable listeners and duplicate test pages. The mean coefficient for all imitations is 0.61 (standard error = 0.01), indicating strong to moderate agreement amongst the reliable listeners [47].

### Ratings between imitations and imitated sounds

Although we did not explicitly inform listeners that the vocalisations were imitations of one of the 6 within-class sounds, the similarity ratings provide an indication of how effectively the drum sounds were imitated, in terms of whether listeners rated the imitated drum sound as being most similar to the imitation. In the following analysis we therefore considered only the highest rated sound from each test page, and calculated the proportion of instances where this was also the imitated sound.

#### Results

Of 1419 completed test pages (excluding unreliable listeners and duplicates), there were 516 instances where the highest rated sound was the ‘target’ (i.e. imitated) sound, and 903 where the target sound did not receive the highest rating. This shows that listeners considered the imitations as most similar to their target sounds at above chance (where chance: 1419/6 = 263.5). This result concurs with similar previous studies on the identification accuracy of vocalisations of everyday sounds [5] and text–based meanings [48], however the overall portion of tests where the target sound received the highest rating was quite low, at 36.4%. A similar figure is observed when duplicates are included, at 36.5%. The method we used to select the drum sounds means that some sounds are more similar than others, and there should be high similarity between certain sounds, where we might expect some confusion between the target and other similar sounds. This appears to be the case: in 856 (60.3%) of the tests the target sound was rated first or second highest.

**Fig 2:**
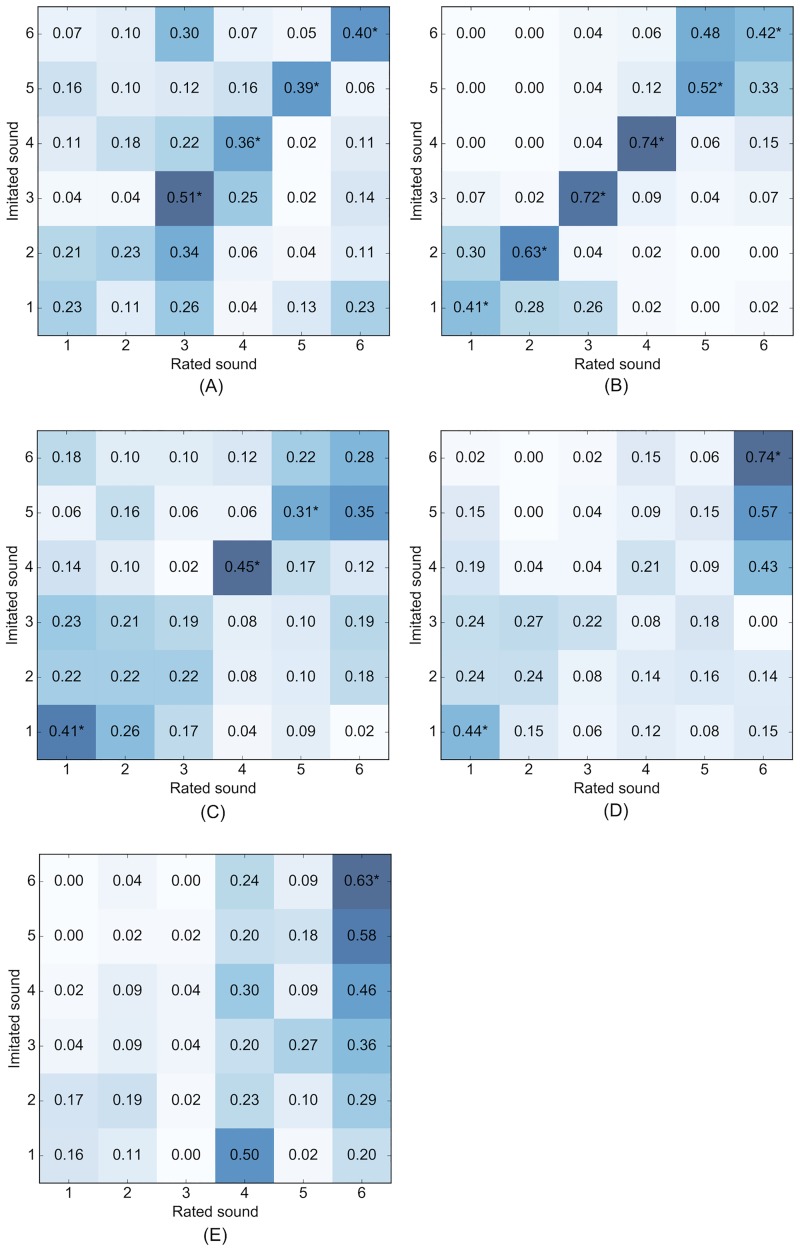
Contingency tables of the highest rated sound for each imitated (i.e. target) sound, by drum category. Cell values and shading indicate the proportion (0–1) of tests for a given imitated sound where the rated sound was considered most similar to the imitation. Asterisks in the diagonals indicate cases where the imitated sound was rated most similar to the imitation, significantly above chance (*p*_*adj*_ <0.05). (A) Cymbals, (B) Hats, (C) Kicks, (D) Snares, (E) Toms.

To investigate the effect of this confusion among the drum sounds, we constructed contingency tables for each drum class (Fig 2), giving the proportion of instances where the target sound was rated highest, for all 30 drum sounds. We conducted a one-way, one-sample *z*–test for proportions on each matching imitated and rated sound pair, i.e. the diagonals in Fig 2, using the *proportions_ztest* function from the *statsmodels* module for Python [49].

The tests within each contingency table were corrected for using the Benjamini and Hochberg false discovery rate correction (FDR = 5%). Of the 30 drum sounds, 16 were imitated such that listeners considered the target sounds as being most similar to the imitations. This is the case for all (six) hats, four cymbals and three kick drums (*p*_*adj*_ <0.05), however, Fig 1D and 1E show that certain sounds in the snare and tom categories were regularly rated highly, irrespective of the sound being imitated. This effect is largest for *snare6*, *tom4* and *tom6*.

#### Discussion

Overall the listeners rated the imitated sound (out of six possible sounds) as most similar to the imitation at above chance, which is an encouraging finding given that the imitations were compared only to same–category sounds. This is even more remarkable given that the imitators were not specifically instructed to imitate the sounds such that they could be differentiated based on the imitations, or made aware that the imitations would be used for the listening test. If we consider this in terms of ‘match rate’ (i.e. the number of tests where the highest rated sound was the imitated sound), we find considerable variation across drum sounds (from 4% to 74%). This is more variable than the findings from [5], where listeners were able to correctly identify target sounds from imitations of everyday and artificial sounds, at rates of 75% and 86% respectively. This is likely due to there being greater similarity between some of our same–category stimuli, and the nature of the task: we did not explicitly ask listeners to identify the imitated sound (as is the case in previous studies on vocal imitation accuracy). We will now discuss the imitation strategies and acoustic characteristics of the imitated sounds that may have contributed to some sounds resulting in a higher ‘match-rate’ than others.

Sounds from the hat category had the highest match rate. Here the greatest confusion was between *hat1*– *hat2*, and *hat5*—*hat6*: *hat1* and *hat2* are both closed hats with very short decay times (0.63s and 0.13s respectively), whereas *hat5* and *hat6* are both open hats with relatively longer decay times (10.27s and 5.69s respectively). This is reflected in the imitations, with mean durations of 0.33s (*hat1*), 0.25s (*hat2*), 3.34s (*hat5*), and 3.18s (*hat6*). This shows that although the duration of the imitations tends to be relative to the duration of the stimulus, the differences are compressed. In addition, the interactions of the top and bottom plates in *hat5* and *hat6* are similar, resulting in similar temporal patterns in the decay. In contrast, *hat3* is a half–open hat, with a unique and distinguishable amplitude envelope compared to the other hat sounds, and *hat4* has a clear, rhythmic repeating pattern in the decay, again giving it a unique temporal signature. It appears listeners did not always consider the target sound as most similar to the imitation when there was similarity in the temporal signature between target and non-target sounds. The differences in temporal features (such as duration and decay shape) are less extreme within the other drum categories (see Table 1), where greater confusion between sounds is observed.

The match rate was similar for the cymbals and kicks, with four and three sounds from each category being rated as most similar to their imitations with above chance accuracy. There was notable confusion between *kick1*, *kick2* and *kick3*: these are all acoustic kick drums with similar resonance patterns and amplitude envelopes. In addition, they are notably brighter than the other kick sounds and have similar short, click–like attack characteristics (whereas the other kick sounds contain considerably less high frequency content). There was also confusion between *kick5* and *kick6*: these are lower in pitch compared to the other kick sounds, and are of a similar duration (0.94s and 0.6s respectively). The mean durations of the imitations from both sounds are similar: 0.4s (*kick5*) and 0.47s (*kick6*), and when listening to the imitations it was apparent that most imitators used similar techniques for both sounds, typically vocalising a stop consonant followed by a voiced decay.

Imitations of all three acoustic snare sounds (*snare4*, *snare5*, *snare6*) were (on average) rated as being most similar to sound *snare6*. This sound contains a more prominent snare rattle compared to *snare4* and *snare5*. Many of the imitators used alveolar, post alveolar or retroflex fricatives to imitate the rattle, and may not have been able to suitably differentiate their vocalisations with respect to the amount of snare rattle they were imitating. It is has been previously demonstrated that imitators will emphasise the salient characteristics of a sound when imitating it [4], therefore it may be that by over–emphasising the snare rattle on any rattling snare drum, the imitators inadvertently made their imitation sound most like the most ‘rattly’ snare sound.

When selecting the stimulus sounds we intentionally chose a wide range of sounds from each category, varying in the degree of similarity to a seed sample. We therefore expected to find little difference in the ratings between similar drum sounds. This is evident for both the hats and kicks (and to some extent the cymbals), however the same effect is not observed for toms. Here we observe a kind of hubness [50] in the perceptual space, where certain drum sounds appear to be perceptually most similar to all imitations within a category. For example, *tom4* and *tom6* are consistently considered as being more similar to imitations, even when they are not the sound being imitated.

This effect may be in part due to differences in the attack portions of the tom drums. For example, *tom4* and *tom6* are from the same drum but played with different beaters (*tom4* = stick, *tom6* = brush—see Table 1). As such, the decay parts of the sounds are very similar, and it is mainly the attack portions that differ: *tom6* contains more noise in the attack portion due to the use of a brush. This difference was not always reflected in the imitations, with 7 of the 14 imitators using affricates to vocalise the attack for both sounds. Furthermore, *tom1* and *tom2* are ‘rim shots’ (where the rim of the drum is struck with the stick), whereas the other tom sounds are not, however many of the imitators (12/14) used the same affricates or stop consonants for the attack portion of these sounds as they did for imitations of the other toms. It is arguably quite difficult to vocalise this rim sound, however there are two imitations for each of *tom1* and *tom2* where the rim sound was imitated using non–pulmonic consonant clicks: for these imitations, the imitated sounds were rated as being most similar when averaging across listeners.

This highlights the importance of imitating the attack characteristics for a sound to be identified, at least for the types of tom sounds used in the present study. Attack time is a well known salient timbral descriptor for instrument or sound discrimination tasks [13], however it has been shown that people tend to not differentiate between subtle differences in attack times when imitating sounds [6]. This presents an interesting problem for modelling similarity between vocalisations and percussive sounds: if imitators apply the same imitation technique to vocalise perceptually different attack times then this descriptor may be of little practical use.

Finally, because the toms are pitched we expected pitch–accurate imitations to be correctly identified, assuming pitch as a salient feature. The *F*_0_ for toms 1–6 is 84, 66, 79, 99, 66 and 102 Hz respectively. Most of the imitations for *tom1* and *tom2* were closest in pitch to the imitated sounds (10/14 for both). Therefore if the listeners based the similarity ratings mainly on pitch, we would expect these imitations to be correctly identified, or else confused with sounds of a similar pitch (the pitch differences between *tom1*–*tom3* and *tom2*–*tom5* are very small). However, the majority of these imitations were rated as being most similar to sounds *tom4* and *tom6*, which are similar in pitch to each other, but considerably different to the other toms. This indicates that the listeners did not necessarily use pitch as a cue for similarity between imitations and pitched percussion sounds.

### Similarity ratings between imitations and imitated sounds

The results presented in the previous section suggest that imitations of certain sounds were more representative of the target sound than others, in terms of listeners giving the highest rating to the target sound. In this section we investigate whether similarity ratings between imitations and target sounds are higher than between imitations and non–target sounds, whether this varies between drum categories, and if the imitator has an effect on the ratings. The responses were analysed using linear mixed effect regression (LMER) with the lme4 package [51] for R [52]. LMER is well suited to this task given that all listeners did not provide ratings for all imitations but only a randomly–selected set of 28 imitations (giving an unbalanced dataset). In addition, it allows us to model the dependencies between ratings for each listener, drum category and imitator.

#### Results

**Fig 3:**
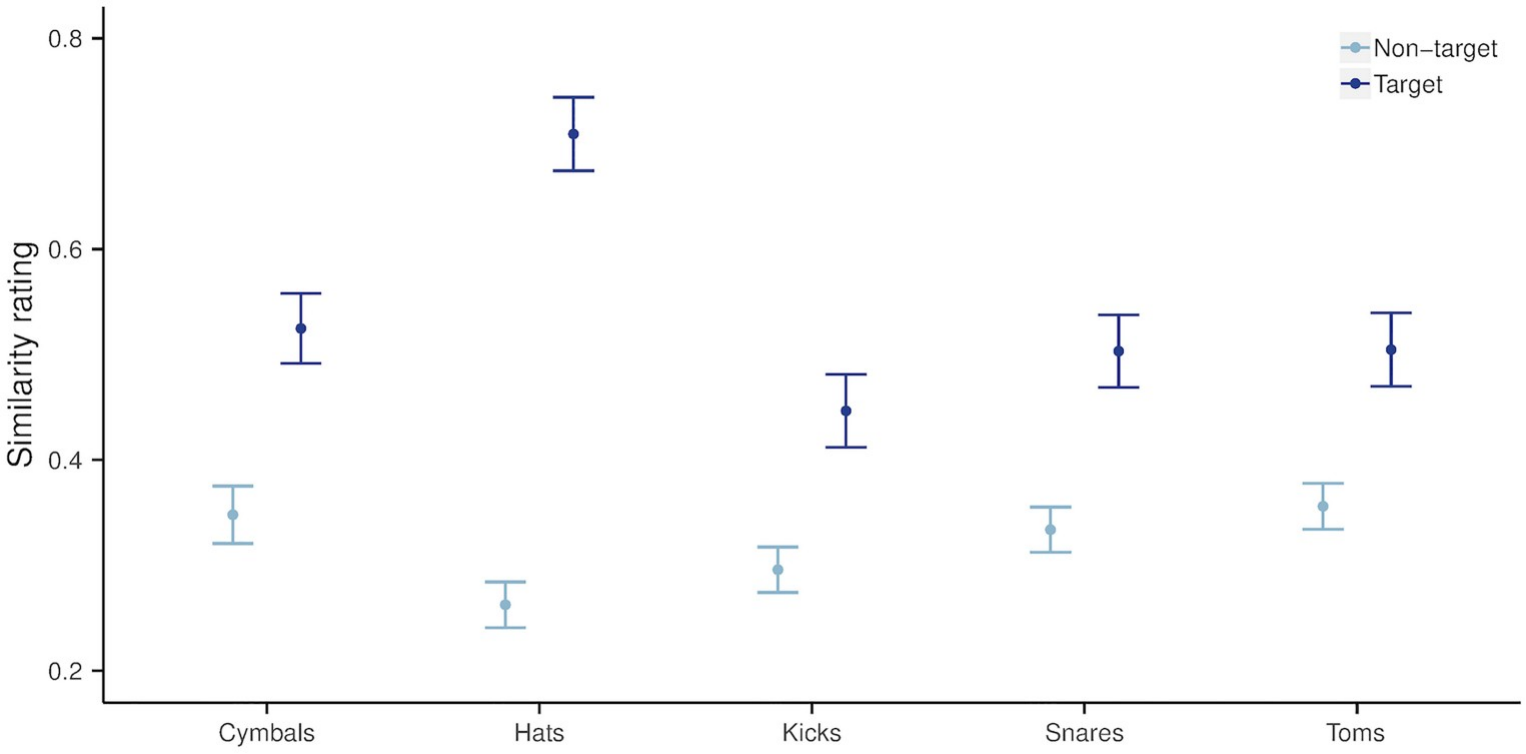
Comparison of similarity ratings between imitations and target vs. non–target sounds, by drum category. Values are mean rating parameter estimates with 95% Wald confidence intervals.

The full model was specified with *rating* as the response variable, fixed effects of *drum category* and *target* (a dummy variable indicating whether the rated sound was the imitated sound) with an interaction term, and random intercepts for *imitator*, and *listener*. Given that the ratings are not independent at the level of each trial, we also included a random intercept for *trial* nested in *listener*, i.e. for the interaction between *listener* and *trial*, where *trial* is a factor variable specifying the index of the test page. The full model specification, as fitted in lme4 is [Rating ∼ Category * Target + (1|Listener/Trial) + (1|Imitator)]. Parameter estimates were then extracted for each combination of the fixed effect levels, and 95% Wald confidence intervals were calculated. The results are given in Fig 3. For each drum category there is a statistically significant difference (*α* < 0.05) between the ratings of target vs. non–target sounds, as indicated by the mean rating estimates and 95% confidence intervals. This difference is largest for hats, and similar for all the other categories.

To establish whether this effect differed between the drum sounds within each category, we modified the above model, replacing the fixed effect of *drum category* with *rated sound*, i.e. [Rating ∼ Sound * Target + (1|Listener/Trial) + (1|Imitator)]. Parameter estimates were extracted for each combination of the fixed effect levels (i.e. ratings for each sound in both target and non-target scenarios), given in Table 2. For 22/30 sounds the rated sound received a significantly higher (*α* < 0.05) similarity rating when being rated against its respective imitation, compared to when rated against an imitation of another sound. For the eight drum sounds where the confidence intervals overlap, the target sound was still rated higher on average than the non–target sounds. This shows that each drum sound received the highest rating when it was rated against its respective imitations, even when it was not considered the sound *most* similar to the imitation (as noted previously). For example, although all the tom sounds received the highest rating when they were compared against a target imitation, the non–target ratings for *tom6* are higher than the target ratings for most of the other tom sounds (Table 2).

**Table 2:** Comparison of similarity ratings between imitations and target vs. non–target sounds, by drum sound. Values are mean rating parameter estimates with 95% Wald confidence intervals (CIs). Cases where the CIs overlap between conditions for each drum sound are given in bold.

Cymbals	1	2	3	4	5	6
*target*	0.53 (0.45, 0.60)	**0.47 (0.39, 0.55)**	0.60 (0.53, 0.68)	0.52 (0.44, 0.60)	0.50 (0.43, 0.58)	**0.53 (0.45, 0.61)**
*non–target*	0.35 (0.31, 0.39)	**0.38 (0.34, 0.43)**	0.44 (0.39, 0.48)	0.32 (0.28, 0.37)	0.19 (0.14, 0.23)	**0.42 (0.37, 0.46)**
Hats	1	2	3	4	5	6
*target*	0.67 (0.58, 0.75)	0.78 (0.70, 0.86)	0.70 (0.61, 0.78)	0.76 (0.68, 0.85)	0.72 (0.64, 0.79)	0.64 (0.56, 0.72)
*non–target*	0.22 (0.17, 0.26)	0.16 (0.11, 0.20)	0.28 (0.23, 0.33)	0.30 (0.25, 0.35)	0.33 (0.28, 0.38)	0.30 (0.25, 0.34)
Kicks	1	2	3	4	5	6
*target*	0.52 (0.44, 0.60)	**0.41 (0.34, 0.49)**	**0.35 (0.27, 0.43)**	0.49 (0.41, 0.57)	0.47 (0.40, 0.55)	0.45 (0.37, 0.53)
*non–target*	0.33 (0.28, 0.37)	**0.37 (0.32, 0.41)**	**0.30 (0.25, 0.34)**	0.19 (0.15, 0.24)	0.31 (0.27, 0.36)	0.28 (0.24, 0.33)
Snares	1	2	3	4	5	6
*target*	0.61 (0.54, 0.69)	0.47 (0.40, 0.55)	0.46 (0.38, 0.54)	**0.40 (0.32, 0.48)**	**0.42 (0.34, 0.50)**	0.66 (0.58, 0.74)
*non–target*	0.36 (0.31, 0.40)	0.32 (0.28, 0.37)	0.22 (0.18, 0.27)	**0.34 (0.29, 0.38)**	**0.38 (0.33, 0.43)**	0.38 (0.34, 0.43)
Toms	1	2	3	4	5	6
*target*	0.48 (0.40, 0.57)	0.40 (0.32, 0.48)	**0.33 (0.25, 0.41)**	**0.57 (0.49, 0.65)**	0.50 (0.42, 0.57)	0.75 (0.67, 0.83)
*non–target*	0.26 (0.21, 0.31)	0.25 (0.20, 0.29)	**0.26 (0.21, 0.30)**	**0.50 (0.45, 0.54)**	0.32 (0.28, 0.37)	0.55 (0.51, 0.60)

**Fig 4:**
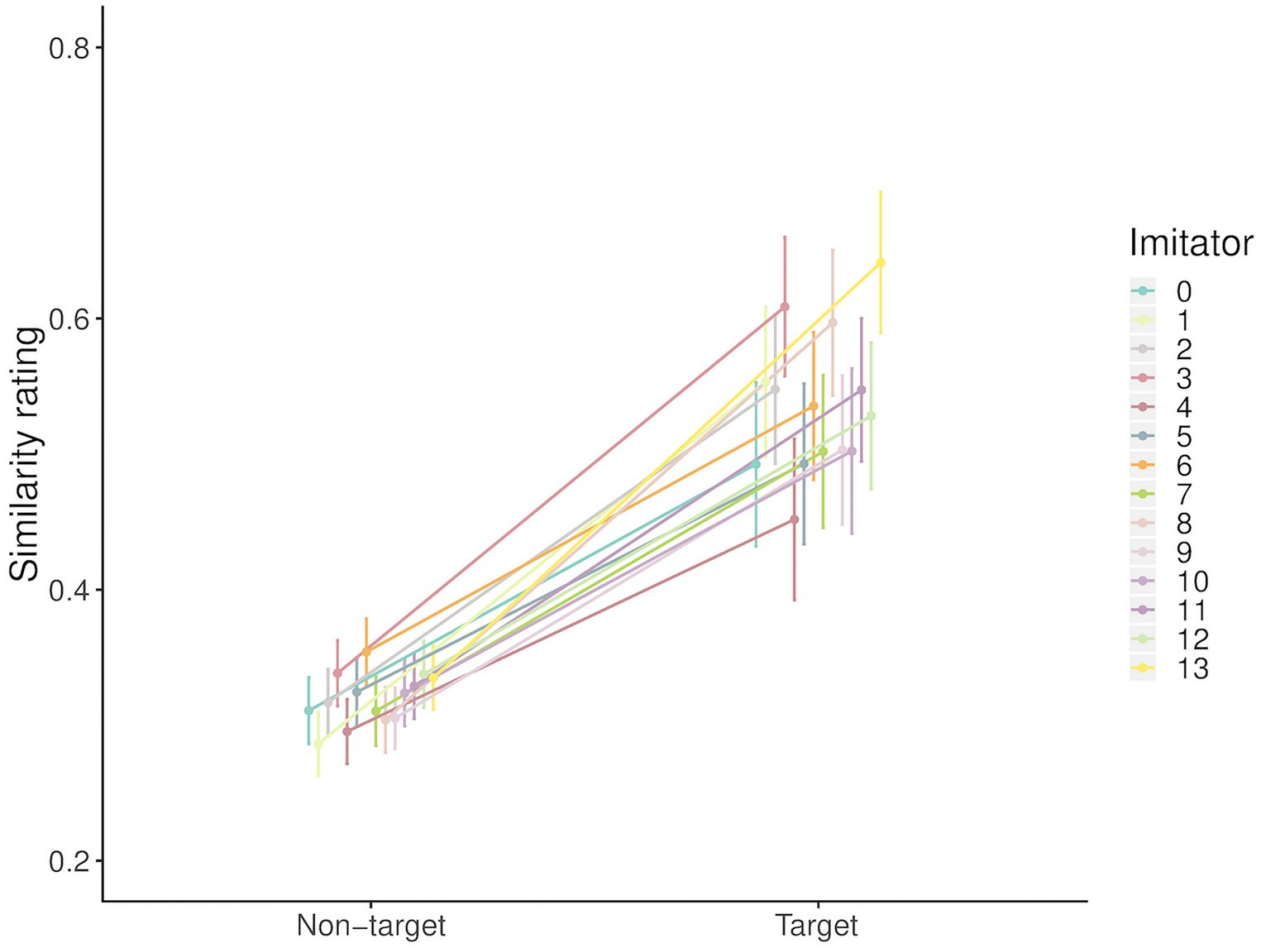
Comparison of similarity ratings between imitations and target vs. non–target sounds, by imitator. Values are mean ratings with 95% Wald confidence intervals.

Finally, we investigated the effect of *imitator* on the ratings, to establish whether certain imitators received higher ratings when the imitation was being rated against the target sound, vs. the non-target sounds. To test this we conducted a likelihood ratio test on two versions of the above specified model, with and without the random intercept for *imitator*. This showed no significant difference between the models (*χ*^2^(1) = 1.72, *p* = 0.19). However, as can be seen in Fig 4, there is more variance between the imitators for the target sounds vs. non-target sounds, with mean ratings ranging from 0.452 and 0.641.

#### Discussion

This analysis compared the ratings for the imitated sound to the *average* ratings for the five same–category sounds that were not imitated. Therefore, even if one or two of the non–imitated sounds were perceptually similar to the imitated sound, the less similar sounds bring down the average rating. Imitators were able to inadvertently identify and imitate the distinguishing characteristics of different sounds within each category, to an extent that enabled listeners to discard some of the sounds as less similar to the imitated sound than others. It is interesting to note that this is also the case where particular sounds were regularly rated as being most similar to any imitation (for example *snare6*, *tom4* and *tom6*). Even when the target sound was not rated highest, it was rarely rated lowly compared to the other sounds. Indeed, the mean reciprocal rank (MRR) for the target sounds across all sounds was 0.58 (where a random ranking would give an MRR of 0.41).

The similarity ratings for target sounds were notably higher for the hats than for the other drum categories (all of which had similar rating values). We found that many of the imitators used speech–related vocal techniques (as used when vocalising fricatives and affricates) to imitate the noisy spectral shape of the hats, and this, combined with the distinctive temporal shapes for each hat sound resulted in higher similarity ratings and identification accuracy (we note that there was less variance in temporal shape between the cymbals, where we also observe lower identification accuracy compared to hats). There is also more contrast in the temporal shapes of the hat sounds compared to all the other drum categories, and this was reflected in the way listeners used the scale (with generally greater contrast between the ratings for target vs. non–target sounds).

Interestingly, the rank order of the similarity ratings was not the same for all tests from a single imitated sound, as might be expected if listeners based the similarity ratings entirely on similarity between the actual drum sounds. Instead, different imitations of the same sounds elicited different rankings. For example, when averaging across listeners, *tom6* was rated as most similar to 10/14 of the imitations of *tom5*. However, in 6/10 of these imitations the second most similar rated sound was *tom5*, whereas it was *tom4* for 4/10. This highlights that the ordering of the drum sounds in terms of similarity ranking with respect to the imitation changed (sometimes considerably) between imitations. It is apparent from listening to the imitations that for a given drum sound the imitation techniques differed between imitators, and also each imitator employed different techniques to imitate the different drum sounds: highlighting specific characteristics of the imitated sounds (such as attack time, pitch, and amplitude envelope). It has been previously shown that specificities in vocal imitations can influence the perceptual similarity between an imitation to it’s referent class [35]. In addition, although there was moderate to strong concordance between listeners’ rankings of the same sounds, listeners may have focussed on different characteristics of the sounds depending on their critical listening experience [53]. Further work is required to establish the factors that may influence between listener differences in this kind of task. It may that the difference in rankings for the same imitated sound is caused by the acoustic characteristics being more or less perceptually relevant to the listeners—for example, the attack time may be most important for one imitation, but for another it may be the amplitude envelope, even when two imitations are of the same sound.

## Model evaluation for perceptual similarity between imitations and imitated sounds

In this section we assess the relationship between the similarity ratings and the drum sound similarity method used to select the samples [40]. This method (herein referred to as PHG) has been shown to be a good predictor of perceptual similarity between same–category drum sounds, therefore we investigate whether it is also suitable for predicting the similarity between vocal imitations and real drum sounds. In addition, we compare the performance of features derived using this method to two baseline feature sets: one based on the spectral envelope, and one using temporal features.

### Method

To recap, in [40] the distance between two sounds is measured as the Euclidean distance between their vectorised spectrograms, constructed with a 93ms window; 11.6ms hop size, Bark scale (72 bins), loudness in dB and scaled using Terhardt’s model for the outer and middle ear [41]. The first baseline feature set is based on Mel frequency cepstral coefficients (MFCCs) [38]. MFCCs essentially provide a smoothed spectral envelope and have been shown to be a good descriptor of timbre [54]. We calculate the first 13 MFCCs for each sound (excluding MFCC 0) with first and second order derivatives, using a 93ms time window and 87.5% overlap. The mean of each MFCC and its derivatives are calculated for each sound, yielding 39 features.

The second baseline feature set (TEMP) consists of seven heuristic temporal features: zero crossing rate (ZCR), log attack time (LAT), temporal centroid (TC), LAT/TC ratio, root mean squared (RMS), temporal crest factor (TCF), and duration. ZCR is the number of times per second the time domain signal crosses zero. LAT and TC are calculated as per the definitions in [40]. RMS is calculated from the amplitude values over the entire time domain signal. TCF is also calculated over the entire time domain signal, and is the maximum amplitude value divided by the RMS.

For a each feature set and drum category, distance was measured between each of the 84 imitations and the six same–category sounds, giving 504 distance values per category, and 2520 in total. We used Euclidean distance in keeping with PHG. Linear mixed effect regression (LMER) models were then fitted for predicting the similarity ratings between the imitations and each of the drum sounds. The model was fitted with *rating* as the response variable, fixed effects of *distance* and *drum sound*, with an interaction term, and random intercepts for *listener*, *imitator*, and *trial* (nested in *listener*). The model was specified in lme4 as [Rating ∼ Distance * Sound + (1|Listener/Trial) + (1|Imitator)].

The interaction term between *distance* and *drum sound* gives a parameter, *β*_*i*_, for each drum sound, *i*, which is an estimate of the slope between *distance* and *rating* for a given sound. Wald 95% confidence intervals (CIs) were then calculated for each of the estimates, *β*_*i*_. For imitated sounds where the upper CI for *β*_*i*_ < 0, we can infer that the slope is significantly below 0 (*α* < 0.05) [55]. In the context of QBV systems, one would expect a useful distance measure to have an inverse relationship with similarity ratings (i.e. more ‘similar’ sounds should be closer in distance than less ‘similar’ sounds, in a given feature space), therefore this measure indicates whether the feature set provides a useful predictor for the imitated sound in question.

The performance of each feature set was evaluated using two metrics: 1) Akaike’s information criterion (AIC), which gives a measure of model fit (note: lower AIC = better model fit); and the percentage of imitated sounds for which *β*_*i*_ is significantly below 0. The slope estimates and their CIs provide a region of expected error around the prediction of the mean *rating* ∼ *distance* slope for a given drum sound, as opposed to the accuracy of predicting the similarity between a new vocalisation and a set of drum sounds, as might be used for QBV. This metric is useful for comparing how well a given feature set performs across drum sounds, however to compare across feature sets, the AIC is a more informative metric. As such, these two metrics are complementary, and an ideal feature set would have a significantly negative *β*_*i*_ for all 30 imitated sounds, whilst still being a relatively good fit to the rating data compared to the other feature sets tested here.

### Results and discussion

The results are given in Table 3. The PHG method performs best, both in terms of AIC and the percentage of sounds where *β*_*i*_ < 0 (53%, corresponding to 16 of the 30 imitated sounds). The temporal features outperform MFCCs, which is interesting given the popularity of MFCCs for many music and speech related tasks. This indicates that the temporal information may be more relevant than the spectral envelope, in terms of how listeners discriminated between the same–category drum sounds tested here. However, PHG implicitly includes both temporal and spectral information, indicating that both types of features are relevant for discriminating between same–category sounds. Indeed, Tindale [56] demonstrated that a combination of temporal and spectral features outperformed temporal features alone, for the task of classifying different snare drums based on playing technique. The PHG method essentially compares two spectrograms, and this makes sense if one considers similar sounds to have similar absolute spectral distributions that evolve similarly over time. It stands to reason that this measure has some merit, because it has been shown that both spectral and temporal information are important for identifying imitated sounds [5], however it has also been shown that imitators tend to transpose spectral features [6, 7], and listeners may not equate such transposition with decreased similarity.

**Table 3:** Performance for each of the feature sets, in terms of i) the LMER model fit (AIC), and ii) percentage of imitated drum sounds for which the rating ∼ distance slope is significantly less than 0 (*α* < 0.05).

Feature set	AIC	% (*β*_*i*_ < 0)
**PHG**	**2375**	**53.3**
MFCC	3161	30.0
TEMP	2684	40.0

Despite performing best out of the feature sets compared here, the overall performance of PHG is still some way from being practically useful for a real world QBV application. When listening to the imitations we found that the imitators made heavy use of vocal techniques from speech: for example using a range of consonants to differentiate attack sounds (in contrast to the participants in [6]), and using diphthongs to emphasise the temporal evolution of resonating pitched sounds. These characteristics and specificities may act as important cues for differentiating between imitated sounds, however it is conceivable that simply comparing the overall shape of two spectrograms does not place suitable weighting on such subtle aspects of the imitations.

**Fig 5:**
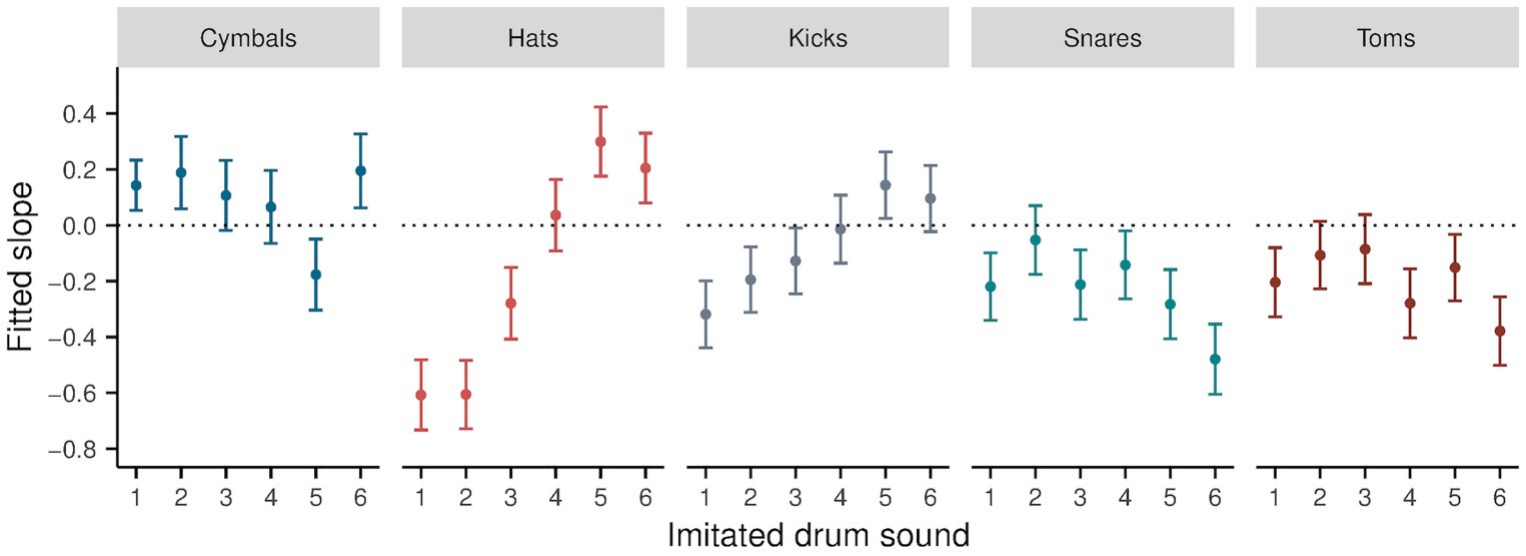
Slope estimates for the LMER model fitted using the distance measure from method PHG. A negative slope indicates a decrease in perceptual similarity with an increase in distance, i.e. sounds for which the method performs well. Values are mean estimates across all imitations for each drum sound, with 95% Wald confidence intervals.

In addition, the prediction performance for PHG varies considerably between the sounds. Fig 5 shows the slope estimates and CIs for all 30 sounds from the LMER model fitted using this method. Interestingly, there does not appear to be any relationship between the identifiability of imitations of particular sounds and how well the acoustic distance measure performs: for example, *hat4*, *hat5* and *hat6* were overall rated as most similar to their imitations with significantly above chance accuracy (Fig 2B), however for these sounds the upper CI of the slope estimates cross 0. The original study describing the measure did not include cymbal or hat sounds, but only kicks, snares and toms [40]. As such it is not clear how suitable the measure is for comparing similarity for cymbal and hat sounds, let alone between imitations and imitated sounds for these drum types.

Finally, we note the slope estimates, although generally below 0, do not approach -1. Listener rating data is inherently noisy, and the concordance amongst listeners varies across the sounds. As such, there will clearly be a glass ceiling for performance, and a perfect model fit would not be useful for a real world application of the LMER model. Indeed, a perfect model fit is not desirable if one is interested in generalisability of the fitted LMER model.

## General discussion and conclusions

We conducted a two part study investigating the imitation of percussion sounds. In the first part, 14 participants imitated 30 drum sounds (six from each of five categories—cymbals, hats, kicks, snares, toms). In the second part, 63 listeners rated the similarity between the imitations and the six same–category sounds. In addition to the individual similarity ratings between imitations and imitated sounds, the experimental design meant that for each imitation, listeners identified the same–category sound that was most similar to the imitation, and a rank ordering of similarity between the imitation and each of the same–category sounds. The main purpose of this study was to establish whether musicians could effectively imitate percussion sounds such that listeners would consider the imitations more similar to the imitated sound than to other same–category sounds. In particular, we were interested in what types of sounds imitators were not able to differentiate, or where listeners exhibited confusion between similar percussion sounds. In addition, we evaluated the performance of an existing measure for perceptual similarity between same–category sounds, when used as a predictor in a linear mixed effect model for perceptual similarity between imitations and same–category sounds.

In general, we found that because some of the same–category sounds were very similar, both the imitation and listening tasks were quite difficult and there was considerable confusion between sounds when the imitators adopted similar vocal techniques to imitate different sounds. The difficulty of the task is highlighted by the fact that 12/63 listeners did not manage to reproduce their own results to a reasonable degree, when given duplicate tasks (based on a rank correlation threshold of 0.5). Nonetheless, there was moderate to strong concordance amongst the (ranked) ratings for the remaining 51 listeners, and for these listeners, 16/30 sounds were overall considered as most similar to their imitations. In addition, even when listeners did confuse percussion sounds, they still rated imitations as being more similar to their respective imitated sounds than the average rating for the other same–category sounds, with a mean reciprocal rank of 0.58 across all trials.

We compared imitations to same–category sounds, many of which have similar spectral distributions. In cases where the spectral distributions did differ, the imitators did not necessarily emphasise these differences, and in some cases were not able to due to the limitations of the vocal production system (for example complex harmonic patterns in tom drum resonances). As such it appears that listeners relied on temporal shape and specificities: sounds with distinctive temporal signatures (such as the hats) were generally more often considered most similar to their imitations than those with congruent temporal envelopes (such as the toms). Spectral features are typically used for measuring timbre, or similarity of percussion sounds, and this highlights an important issue with QBV for percussion sounds: the types of features used in tasks such as drum category classification may not be suitable to measure same–category similarity, particularly when the spectral differences are not easily differentiated using the voice.

Finally, we showed that the auditory image based similarity measure from [40] is a relatively good predictor of the similarity ratings, outperforming both MFCCs and a set of temporal features. Whilst this indicates that the measure may be useful for certain types of sounds, it demonstrates that further work is required to improve its generalisability to a wider set of percussion sounds. Furthermore, recent work on extracting acoustic features using deep learning techniques has demonstrated considerable performance improvements over heuristic descriptors such as MFCCs for QBV [10, 11]. We propose applying such techniques to the dataset presented here. However, we note that the imitators used different techniques to imitate the same sounds, and in some cases the imitators did not vary their technique when vocalising different same–category sounds. To concur with Lemaitre et al. [35], it may therefore be misguided to apply a ‘one size fits all’ solution for measuring similarity between vocalisations and non–vocal sounds. Instead, percussion based QBV systems may benefit from some knowledge of both user–specific and sound–specific imitation strategies and techniques.

The percussion sounds, vocal imitations and listening study data have been made available for further research and reproducibility [57].
