# Correction: Vocal imitation of percussion sounds: On the perceptual similarity between imitations and imitated sounds

**DOI:** 10.1371/journal.pone.0221722

**Published:** 2019-08-22

**Authors:** Adib Mehrabi, Simon Dixon, Mark Sandler

The affiliations for the second and third authors are incorrect. Simon Dixon and Mark Sandler are not affiliated with #1 but with #2: School of Electronic Engineering and Computer Science, Queen Mary University of London, London, England.

## References

[1] Mehrabi A, Dixon S, Sandler M (2019) Vocal imitation of percussion sounds: On the perceptual similarity between imitations and imitated sounds. PLoS ONE 14(7): e0219955 10.1371/journal.pone.0219955 31344080 PMC6657857

